# Renal Cyst’s Dark Secret: A Rare Case of Fungal-Infested Necrotizing Granulomatous Inflammation in a Renal Cyst

**DOI:** 10.7759/cureus.85686

**Published:** 2025-06-10

**Authors:** Punith Jain R, Suryaram Aravind, Barathi Gunabooshanam, Velmurugan Palaniyandi, Hariharasudhan Sekar, Sriram Krishnamoorthy

**Affiliations:** 1 Urology and Renal Transplantation, Sri Ramachandra Institute of Higher Education and Research, Chennai, IND; 2 Pathology, Sri Ramachandra Institute of Higher Education and Research, Chennai, IND

**Keywords:** candida, decortication, granulomatous inflammation, immuno-competent, renal cyst

## Abstract

Fungal infections of renal cysts are exceptionally rare, particularly in immunocompetent individuals, and are often misdiagnosed due to nonspecific clinical and radiological findings. This report presents the first documented case of necrotizing granulomatous inflammation caused by Candida species within a renal cyst in an immunocompetent host. Here we present a 49-year-old immunocompetent male who presented with right loin pain and fever. He had a prior episode of renal cyst infection managed with percutaneous catheter drainage (PCD) three months earlier. Laboratory investigations revealed leukocytosis and marginally elevated serum creatinine. Imaging showed a thick-walled lower pole renal cyst (247 cc) with internal echoes, parenchymal thinning, and features suggestive of chronic obstruction. Initial management involved the use of empirical broad-spectrum antibiotics and ultrasound-guided aspiration with a 12 Fr pigtail catheter placement, yielding 180 mL of turbid fluid. Microbiological cultures, including bacterial and fungal, were negative. Due to persistent drainage and residual cavity on follow-up imaging, the patient underwent open cyst decortication with DJ stent removal. Histopathological analysis confirmed necrotizing granulomatous inflammation with fungal hyphae, consistent with Candida infection. Postoperatively, the patient was treated with oral fluconazole for six weeks. At two-month follow-up, he remained asymptomatic with no signs of recurrence. This case underscores the importance of considering fungal etiologies in persistent or recurrent renal cyst infections, even in immunocompetent patients. Histopathological confirmation is essential when cultures are inconclusive. Combined surgical and antifungal management remains key to achieving definitive resolution.

## Introduction

Fungal infections of the kidney are rare in immunocompetent individuals. Most cases are reported in immunocompromised patients, such as those with diabetes, malignancies, or immunosuppressive therapy [1]. Fungal renal cyst infections are exceedingly rare in immunocompetent individuals, and we share our experience with one such case. The classical signs and symptoms of infection are often absent in these patients, resulting in delayed diagnosis and treatment. However, emerging literature underscores the importance of maintaining a high index of suspicion, as fungal infections can occur even without overt immunosuppression [2,3].

Renal cysts are commonly encountered in clinical practice, and most remain asymptomatic. Infrequently, however, these cysts may become infected and lead to significant morbidity. The case we present involved a thick-walled, infected cyst that contributed to considerable clinical symptoms. It is imperative to recognize such atypical presentations early, as prompt diagnosis and appropriate intervention can prevent complications.

Typically, renal cysts are managed laparoscopically. However, a more aggressive approach may be warranted in the presence of suspicious features, such as wall thickening, internal debris, or atypical imaging findings. A high degree of clinical vigilance is essential to distinguish these lesions from typical benign cysts and to plan for definitive open surgical excision, thereby minimizing the risk of recurrence. Moreover, though rare, certain cystic lesions may harbor malignant potential. This possibility further reinforces the need for comprehensive evaluation and individualized surgical planning in cases that deviate from the expected clinical or radiological patterns.

In patients with renal cyst infection due to fungal aetiology, urine and blood cultures often yield negative results, as fungal organisms tend to be sequestered within the cyst wall, limiting their detectability in routine cultures [4]. Cross-sectional imaging, such as contrast-enhanced computed tomography (CECT) and magnetic resonance imaging (MRI), aids in assessing the anatomical extent of disease but cannot reliably differentiate between bacterial and fungal etiologies. These findings may mimic infarcts, abscesses, or malignancies [5]. Therefore, invasive procedures like percutaneous aspiration may be necessary to identify fungal elements through cytology or histopathology, although these too may fail to isolate the pathogen consistently. Ultimately, definitive diagnosis relies on histopathological examination and tissue culture [6].

Delayed recognition and intervention can result in sepsis, renal dysfunction, and increased morbidity. Early histopathological confirmation, timely surgical management, and appropriate antifungal therapy form the cornerstone of treatment. This case report highlights the variable presentation, diagnostic difficulties, and therapeutic challenges associated with this rare but significant clinical entity.

## Case presentation

Clinical presentation and evaluation

A 49-year-old gentleman presented with right loin pain and fever. He denied lower urinary tract symptoms (LUTS), hematuria, weight loss, or loss of appetite. There was no history suggestive of immunosuppression or tuberculosis. Notably, he experienced a similar episode three months earlier, during which imaging revealed a right renal cyst. He underwent percutaneous catheter drainage (PCD), which was removed after 10 days, and reported symptomatic relief until the current presentation. On examination, the patient was afebrile with stable vitals. Abdominal examination revealed tenderness in the right loin without a palpable mass. Examination of the external genitalia, digital rectal examination (DRE), spine, and other systemic assessments, including cardiovascular, respiratory, and central nervous systems, was unremarkable.

Table 1 illustrates the laboratory investigations in detail. The patient had an elevated total leukocyte count (16,390 cells/cu mm) and an elevated serum creatinine (1.3 mg/dL). Urine microscopy revealed pyuria. However, other blood and urine parameters were within normal limits (Table 1).

**Table 1:** Baseline haematological, biochemical, and microbiological investigations All values are from the initial evaluation prior to definitive surgical intervention. Reference ranges are based on institutional laboratory standards. HPF — High-power field; HbA1c — Glycated haemoglobin;  No Growth / Sterile - no microbial growth observed after incubation.

Parameter	Patient Value	Units	Reference Range
Haemoglobin	13.3	g/dl	13.0 – 17.0
Total Leucocyte Count	16,390	cells/mm3	4,000 – 11,000
Platelet Count	235,000	cells/mm3	150,000 – 450,000
Random Blood Sugar	116	mg/dl	< 140
HbA1c	5.6	%	< 5.7
Blood Urea Nitrogen	11	mg/dl	6 – 20
Serum Creatinine	1.3	mg/dl	0.7 – 1.2
Sodium	138	mEq/L	136 – 145
Potassium	3.7	mEq/L	3.5 – 5.1
Chloride	100	mEq/L	98 – 107
Bicarbonate	22	mEq/L	22 – 29
Urine Pus Cells	100 – 150	/HPF	0 – 5
Gram Stain (Urine)	No organisms seen	-	No organisms / Sterile
Urine Culture	No Growth	-	No Growth / Sterile
Blood Culture	No Growth	-	No Growth / Sterile

**Figure 1:**
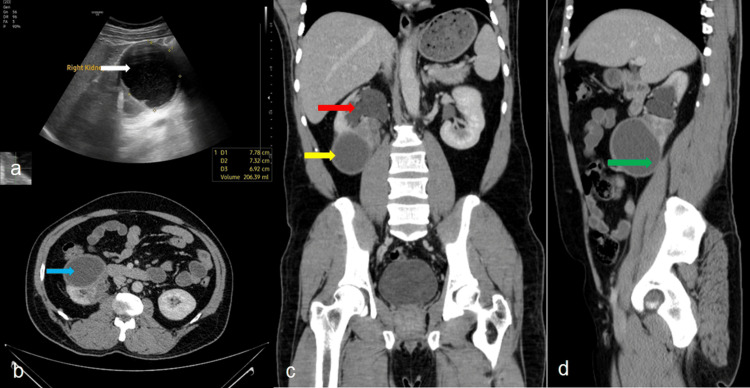
Multimodal imaging of the right renal cyst (a) Ultrasound showing a thick-walled cortical cyst in the lower pole of the right kidney. (b) Axial non-contrast CT depicting a well-defined hypodense lesion with a thickened wall (blue arrow) in the lower pole of right kidney. (c) Coronal contrast-enhanced CT demonstrates a large cyst (yellow arrow) with subtle wall enhancement, cortical thinning, and dilated pelvicalyceal system (red arrow), suggesting chronic obstruction. (d) Sagittal contrast-enhanced CT showing posterior abutment of the cyst to the psoas muscle and compression of the upper ureter (green arrow).

Figure 1 illustrates the right renal cyst's ultrasound and computed tomography (CT) imaging findings. Ultrasonography (Figure 1a) revealed a thick-walled (5 mm) cyst in the lower pole of the right kidney, measuring 8.5 × 8.0 × 7.2 cm, with internal floating echoes suggestive of intracystic debris. Non-contrast axial CT (Figure 1b) confirmed a well-defined, hypodense lesion with a thickened wall. Contrast-enhanced coronal CT (Figure 1c) showed a large cystic lesion with an estimated volume of 247 cc, accompanied by diffuse cortical thinning and reduced parenchymal enhancement, indicative of chronic obstruction. The pelvicalyceal system was dilated. Sagittal CT imaging (Figure 1d) demonstrated posterior extension of the cyst abutting the right psoas muscle, anterior contact with the hepatic flexure, medial proximity to the inferior vena cava (IVC), and extrinsic compression of the upper ureter. The remainder of the right ureter and urinary bladder appeared unremarkable.

Treatment

**Figure 2:**
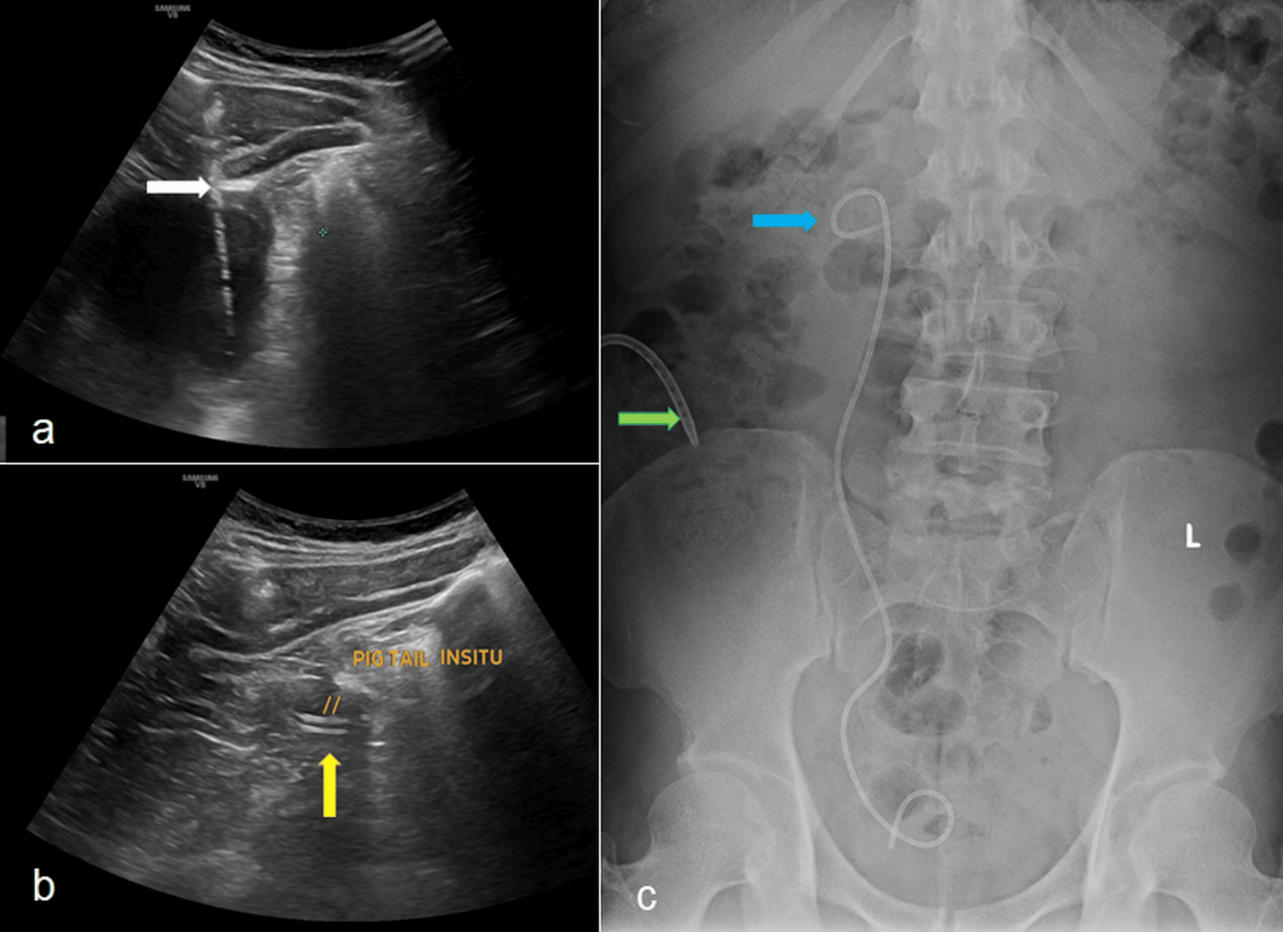
Minimally invasive interventions for renal cyst management (a) Ultrasound-guided aspiration of the renal cyst (white arrow) followed by a percutaneous pigtail catheter placement. (b) Ultrasound confirming correct positioning of the catheter tip within the collapsed cyst cavity (yellow arrow). (c) Plain abdominal radiograph (X-ray kidney, ureter, bladder (KUB)) showing the pigtail catheter in situ (green arrow) and a right-sided double-J ureteral stent (blue arrow).

The patient was initiated on empirical broad-spectrum antibiotics along with supportive care. He subsequently underwent cystoscopy with right retrograde pyelography (RGP) and double-J (DJ) stenting, followed by ultrasound-guided percutaneous pigtail catheter insertion for combined internal and external drainage of the renal cyst (Figure 2). Figure 2a and Figure 2b illustrate the ultrasound-guided placement of a 12 Fr pigtail catheter, through which approximately 180 mL of turbid fluid was aspirated from the cyst cavity. Post-procedure, the patient experienced symptomatic relief by postoperative day one, and a follow-up X-ray of kidney, ureter, and bladder (KUB) confirmed appropriate placement of both the pigtail catheter and the DJ stent (Figure 2c). Microbiological analysis of the aspirated cyst fluid, including gram stain and bacterial culture, fungal stain, and acid-fast bacilli (AFB) testing, revealed no bacterial, fungal, or acid-fast organisms. Cytological examination was negative for malignant cells. At the two-week follow-up, the pigtail catheter continued to drain approximately 25 mL of fluid daily.

Definitive management and outcome

Follow-up imaging demonstrated a significant reduction in the cystic collection and the associated pelvicalyceal system (PCS) dilatation. However, in view of persistent drainage and incomplete resolution of the lesion, a decision was made to proceed with definitive surgical management. The patient underwent routine preoperative optimisation, including adequate hydration and anaesthetic clearance.

**Figure 3:**
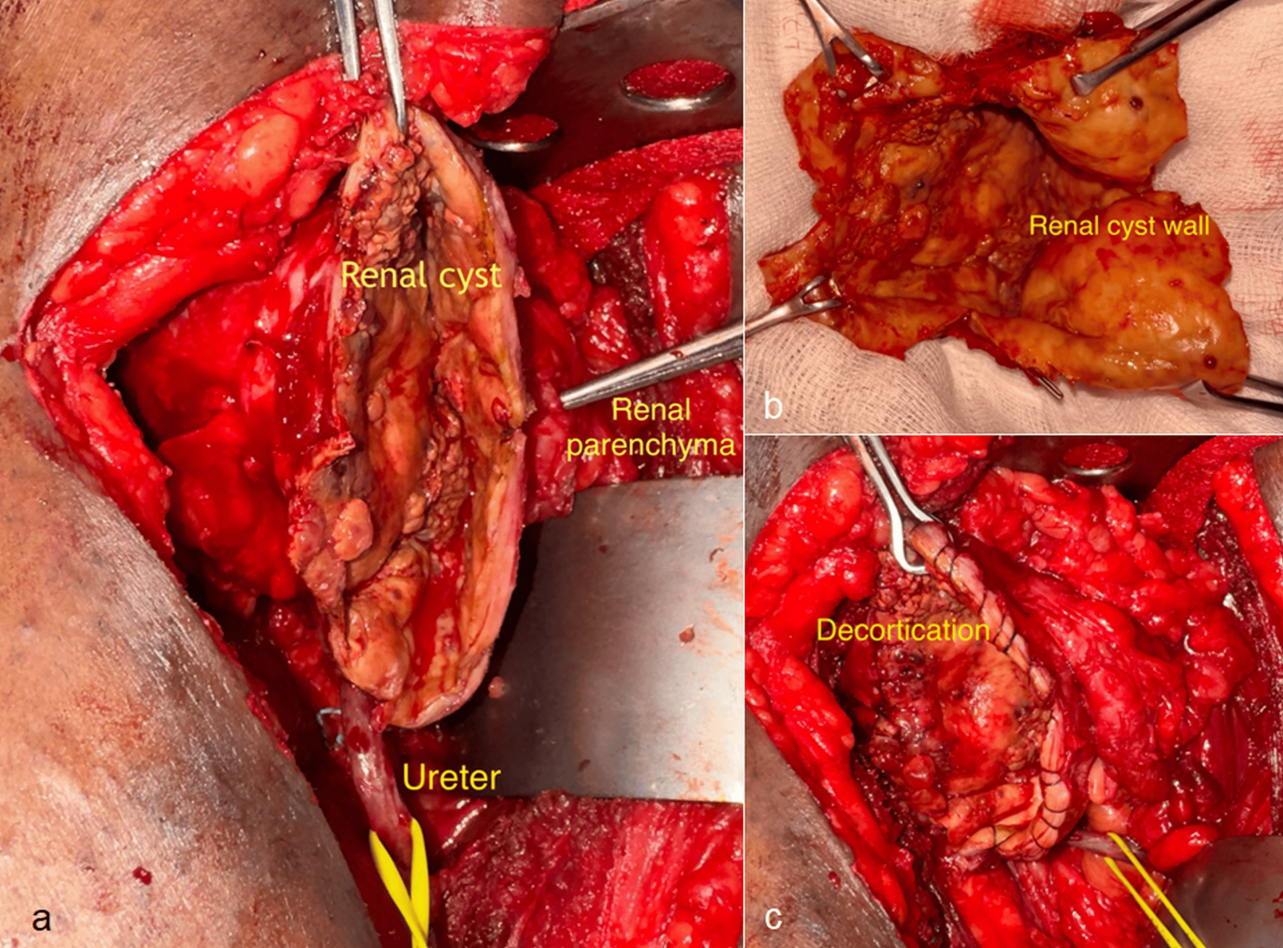
Intraoperative steps of open right renal cyst decortication (a) The renal cyst wall is dissected and carefully isolated from the ureter and surrounding attachments. (b) Excision of the thick, necrotic cyst wall, which appeared inflamed and non-viable. (c) Completed decortication showing no communication with the renal collecting system, and no injury to the ureter or the underlying renal parenchyma.

He subsequently underwent open right renal cyst decortication and DJ stent removal under general anaesthesia and strict aseptic precautions. Figure 3a, 3c illustrates the intra-operative steps of open right renal cyst decortication, and the important structures in relation to the cyst are labelled within for better understanding. The postoperative period was uneventful, and the patient recovered well. The Foley catheter was removed on postoperative day one, and the intra-abdominal drain was removed on day two. The patient remained clinically stable, with no signs of infection or urinary leak. He was discharged in good general condition on postoperative day three with instructions for follow-up evaluation.

**Figure 4:**
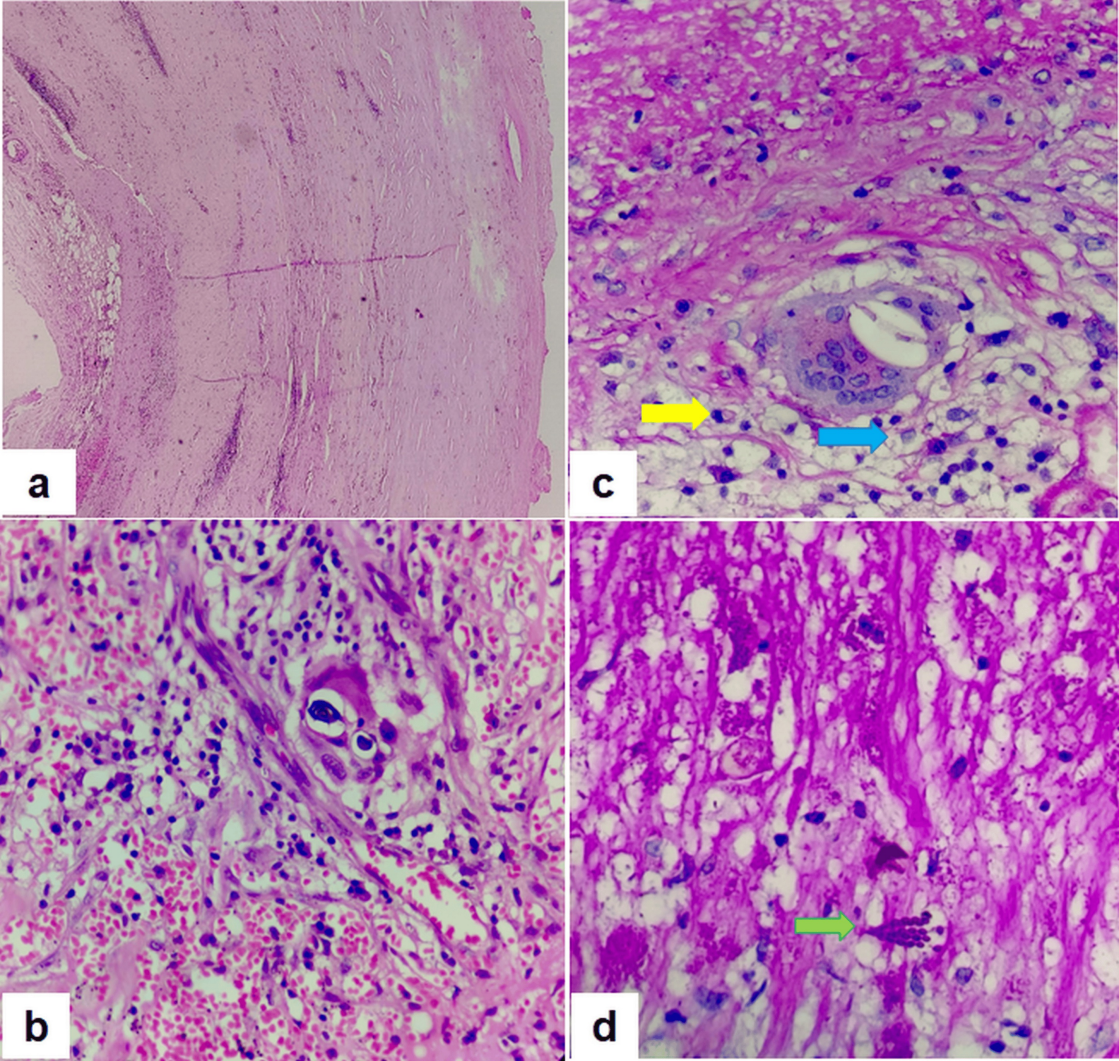
Histopathological examination of the renal cyst wall (a) H&E, 40X: Cyst wall showing vascular congestion and chronic inflammatory infiltrate in the subepithelial region. (b) H&E, 100X: Granulomatous inflammation with multinucleated giant cells and intracytoplasmic fungal yeast forms. (c) H&E, 400X: Budding yeast cells (yellow arrow) and branching pseudohyphae (blue arrow) within fibrous stroma and inflammatory infiltrate. (d) PAS stain, 400X: Pseudohyphae of Candida species highlighted within necrotic tissue (green arrow). H&E - Hematoxylin and Eosin staining, PAS - Periodic Acid-Schiff Staining.

Figure 4 illustrates the histopathological features of the excised renal cyst wall. In Figure 4a, the cyst wall demonstrates vascular congestion and a dense chronic inflammatory infiltrate in the subepithelial region, indicating a longstanding inflammatory response on 40x magnification with hematoxylin and eosin staining. On 100X magnification with hematoxylin and eosin staining (Figure 4b), the presence of ill-defined epithelioid granulomas with multinucleated giant cells, along with intracytoplasmic fungal yeast forms, was noted, findings suggestive of granulomatous inflammation consistent with a fungal etiology, most likely Candida. In Figure 4c (H&E, 400X), the fungal organisms are more clearly visualized, with budding yeast cells (yellow arrow) and branching pseudohyphae (blue arrow) embedded within fibrous stroma, accompanied by chronic inflammatory infiltrate on 400X magnification with hematoxylin and eosin staining. Figure 4d (Periodic Acid-Schiff stain, 400X) confirms the fungal origin, highlighting the pseudohyphae of Candida species (green arrow) within areas of necrotic tissue. Together, these findings support the diagnosis of necrotizing granulomatous inflammation due to Candida infection in the renal cyst wall. The patient was initiated on oral fluconazole therapy for six weeks and followed under close clinical surveillance. At two-month follow-up, he remained asymptomatic with no clinical or radiological evidence of recurrence.

## Discussion

Fungal infections of renal cysts are rare, especially in immunocompetent individuals [7]. Candida species are the most frequently implicated pathogens, While most cases occur in immunocompromised hosts, such as those with diabetes, malignancies, or undergoing immunosuppressive therapy [8,9]. Although Candida infections are more common in immunocompromised patients, their occurrence in immunocompetent individuals remains exceptional. This case highlights a rare instance of an infected renal cyst caused by Candida in an immunocompetent patient, emphasizing the diagnostic challenges, management complexities, and recurrence risks associated with such infections.

Diagnostic challenges

Fungal renal cyst infections are often misdiagnosed due to a nonspecific clinical presentation [10]. Symptoms such as fever, loin pain, and constitutional complaints overlap with bacterial infections, often leading to an initial empirical antibiotic approach. Routine urine and blood cultures fail to identify fungal pathogens, as fungi tend to localize within cystic structures without systemic dissemination [11,12]. Imaging modalities, including contrast-enhanced CT, may reveal thick-walled, multiloculated cysts with subtle enhancement, but these findings are not pathognomonic. In our case, the cyst was found to abut adjacent structures, with associated pelvicalyceal system (PCS) dilatation, suggesting extrinsic compression. However, a definitive diagnosis was made after histopathological examination, which revealed necrotizing granulomatous inflammation with fungal hyphae, confirming Candida as the causative organism.

Management complexities

Managing fungal renal cyst infections is challenging, particularly in determining the balance between conservative and surgical interventions. While percutaneous catheter drainage (PCD) is often the first step, it may not always achieve complete resolution. In our case, PCD provided symptomatic relief but was inadequate in eliminating the infection, as residual cystic fluid persisted. The recurrence of symptoms and persistent lesion on follow-up imaging necessitated surgical intervention in the form of open cyst decortication [13]. Surgical decortication is the definitive treatment for refractory or recurrent fungal infections, especially when there is associated obstruction or parenchymal involvement. Adjuvant antifungal therapy is critical in managing these infections. The choice of antifungal is based on the isolated organism's drug susceptibility. Due to their favourable oral bioavailability and renal penetration, azole antifungals, particularly fluconazole, are the first-line agents for Candida infections [14]. A six-week course of fluconazole was initiated postoperatively in our patient, ensuring the eradication of residual fungal elements and preventing recurrence.

Recurrence risk without surgery

The recurrence of fungal renal cyst infections without definitive surgical management is well-documented [15]. Persistent infections despite prolonged antifungal therapy are common when surgery is deferred, particularly in cases with cyst-associated urinary obstruction. Chronic suppressive antifungal treatment may be used in select cases where surgery is not feasible, but its long-term efficacy remains uncertain. In our case, the need for surgical excision was highlighted by the persistent infection despite drainage, emphasizing the importance of timely intervention to prevent chronic renal dysfunction and recurrence [16].

This is the first reported case of necrotizing granulomatous inflammation caused by Candida species in a renal cyst in an immunocompetent individual, confirmed through histopathological analysis after surgical decortication. This case is unique due to the absence of conventional risk factors such as diabetes, malignancy, or immunosuppressive therapy, as well as the diagnostic complexity, standard urine and blood cultures were negative, and imaging features were nonspecific, mimicking bacterial cyst infection. The identification of fungal hyphae in necrotizing granulomatous tissue underscores the importance of maintaining a high index of suspicion and pursuing histopathological evaluation when clinical improvement is lacking despite appropriate antimicrobial therapy. This report expands the clinical spectrum of renal fungal infections and supports the role of early surgical intervention and tissue diagnosis in guiding definitive management.

## Conclusions

Fungal infection of a renal cyst in an immunocompetent individual is exceedingly rare and diagnostically challenging due to nonspecific clinical and radiological features. This case highlights the critical role of histopathology in establishing a definitive diagnosis, especially when conventional cultures are negative. Persistent or recurrent cystic infections unresponsive to antibiotics should prompt consideration of fungal aetiology. Early surgical intervention, combined with targeted antifungal therapy, is essential for complete resolution and prevention of recurrence. This report underscores the need for heightened clinical suspicion and individualized treatment strategies in managing atypical renal cyst infections.
